# Systematic review of clinical practice guidelines recommendations about primary cardiovascular disease prevention for older adults

**DOI:** 10.1186/s12875-015-0310-1

**Published:** 2015-08-20

**Authors:** Jesse Jansen, Shannon McKinn, Carissa Bonner, Les Irwig, Jenny Doust, Paul Glasziou, Brooke Nickel, Barbara van Munster, Kirsten McCaffery

**Affiliations:** Screening & Test Evaluation Program (STEP), Sydney School of Public Health, The University of Sydney, Edward Ford Building A27, Sydney, NSW 2006 Australia; Centre for Medical Psychology and Evidence-based Decision-making (CeMPED), The University of Sydney, Edward Ford Building A27, Sydney, NSW 2006 Australia; Faculty of Health Sciences and Medicine, Bond University, Robina, QLD 4226 Australia; Academic Medical Centre, Department of Internal Medicine, University of Amsterdam, Meibergdreef 9, 1105 AZ Amsterdam, Netherlands; Department of Geriatrics, Gelre Hospitaal, Albert Schweitzerlaan 31, 7334 DZ Apeldoorn, Netherlands

**Keywords:** Aging, Cardiovascular diseases, Guidelines, Shared decision making

## Abstract

**Background:**

Clinical care for older adults is complex and represents a growing problem. They are a diverse patient group with varying needs, frequent presence of multiple comorbidities, and are more susceptible to treatment harms. Thus Clinical Practice Guidelines (CPGs) need to carefully consider older adults in order to guide clinicians. We reviewed CPG recommendations for primary cardiovascular disease (CVD) prevention and examined the extent to which CPGs address issues important for older people identified in the literature.

**Methods:**

We searched: 1) two systematic reviews on CPGs for CVD prevention and 2) the National CPG Clearinghouse, G-I-N International CPG Library and Trip databases for CPGs for CVD prevention, hypertension and cholesterol. We conducted our search between April and December 2013. We excluded CPGs for diabetes, chronic kidney disease, HIV, lifestyle, general screening/prevention, and pregnant or pediatric populations. Three authors independently screened citations for inclusion and extracted data. The primary outcomes were presence and extent of recommendations for older people including discussion of: (1) available evidence, (2) barriers to implementation of the CPG, and (3) tailoring management for this group.

**Results:**

We found 47 eligible CPGs. There was no mention of older people in 4 (9 %) of the CPGs. Benefits were discussed more frequently than harms. Twenty-three CPGs (49 %) discussed evidence about potential benefits and 18 (38 %) discussed potential harms of CVD prevention in older people. Most CPGs addressed one or more barriers to implementation, often as a short statement. Although 27 CPGs (58 %) mentioned tailoring management to the older patient context (e.g. comorbidities), concrete guidance was rare.

**Conclusion:**

Although most CVD prevention CPGs mention the older population to some extent, the information provided is vague and very limited. Older adults represent a growing proportion of the population. Guideline developers must ensure they consider older patients’ needs and provide appropriate advice to clinicians in order to support high quality care for this group. CPGs should at a minimum address the available evidence about CVD prevention for older people, and acknowledge the importance of patient involvement.

**Electronic supplementary material:**

The online version of this article (doi:10.1186/s12875-015-0310-1) contains supplementary material, which is available to authorized users.

## Background

### Available evidence for older people

It is a major challenge for clinicians to provide appropriate and patient centered care for older people with comorbidities. Clinical practice guidelines (CPGs) aim to support clinician decision making, however, CPGs may not always be straight forward to implement for older people for several reasons [1]. Older people have generally been excluded from clinical trials, and when included they are generally more fit and healthy than the older people in the community [2, 3]. Furthermore, clinical trials often do not address outcomes that may have high priority for older adults (e.g. quality of life and independent living) [4]. Moreover, most CPGs focus on a single disease, however the prevalence of comorbidities increases with age and studies in Australia [5] and Scotland [6] estimate that around 70 percent of people over 75 have two or more chronic conditions. Applying CPGs for each condition leads to polypharmacy, increases treatment burden, and risk of adverse events [1].

### Barriers to implementation of CPGs

There are other barriers to applying CPGs to older people. Treatment in older populations is generally more complex due to comorbidities and associated polypharmacy. Data from the US and Australia suggest that over 90 % of older persons (75 years and above) take one or more prescription medication, with approximately 40 % of older persons using five or more prescription medications at one time [7, 8]. Older people who take multiple medications are at increased risk of experiencing adverse drug reactions [9] and the presence of comorbidities and polypharmacy also means that it can be difficult to predict the effect of a treatment, and compare the overall benefits and harms [10]. Moreover, older people are heterogeneous in terms of general health status, frailty and cognitive function and even older people with the same diagnosis may therefore respond different to treatment [4]. Older people will also vary in terms of prognosis, and when applying CPG recommendations to older people it is important to determine if the person is likely to benefit from the medication within their remaining life (time needed to treat to benefit) [11, 12]. Moreover, they will vary in their treatment- and health outcome preferences (e.g. length of life versus quality of life, physical and cognitive functioning, risk reduction, tolerance of side effects) [13–15].

### Tailoring treatment to older patient context, preferences and goals

Decision making about initiating treatment in older people therefore requires careful tailoring to the individual patient’s circumstances and it is critical to involve the older patient and take their preferences into account in the shared decision. Older patients’ preferences and treatment goals are are more variable than younger people, and likely to change depending on  factors such as health and mood [15, 16]. This highlights the importance of eliciting patient preferences when deciding on treatment in this group. Most people, including older people [17], prefer to be involved in the decision making process. The majority of older people want to discuss options and receive information even though they may not wish to be involved in making the final decision [18] and value the input of their family member/carer who are often present in the consultation [19]. However, CPGs are often not set up in a way that optimally supports patient involvement and shared decision making [20], this is a general limitation although it is probably particularly relevant for older people.

### Need for guidance primary CVD prevention in older people

In this paper we focus on CPGs for primary cardiovascular disease (CVD) prevention in older people. Worldwide, the population is aging and because CVD risk increases with age, this creates a major public health burden. Moreover, medication use for CVD prevention is common in older people, for example one-third of people aged 75–84 in Sweden [21] are treated with statins and in the US over 80 % of adults with hypertension aged 60 or older receive anti-hypertensive medication [22]. This means that there is a clear imperative for CPG for primary CVD prevention to address older people. Most international cardiovascular disease (CVD) primary prevention CPGs encourage the use of 5- or 10 year absolute (or overall, global or combined) CVD risk scores (AR) to target preventive treatment in asymptomatic patients who are at high risk [23]. However, CVD risk prediction models are not well validated in older people, for example the widely used Framingham risk equation is based on a patient cohort with an upper age of 74 [24]. Because AR increases with age, and people at higher risk benefit more in terms of risk reduction, it is reasonable to argue that older people (and especially otherwise healthy older people) have the potential to benefit at least as much from primary CVD prevention as younger people [25, 26] and otherwise healthy older people should not be denied potentially effective preventative medication based on their age alone. However, although there is some evidence of the benefits of blood pressure [27, 28] and cholesterol lowering medication in older people [29, 30], most of these studies did not take into account comorbidities [31]. Also, the harms of medication (e.g. myopathy with cholesterol medication and risk of falls with blood pressure medication) are more likely to occur in older people [31–34] and older people are likely to vary widely in the relative importance they place on benefits and harms of CVD medication [15]. Moreover, not every older patient will be able to achieve benefits from long-term preventative CVD medication during their remaining life span [12].

In summary, when making decisions about primary CVD prevention for older people, clinicians have the challenging task of weighing AR with life expectancy, co-morbidities, the benefits and harms of medication, and preferences of patients and family members/carers. Not surprisingly, clinicians have reported the need for clearer guidance in this area [35]. The purpose of this paper is to systematically review international CPGs for primary CVD prevention to examine the extent to which they address older adults and take into account factors important for patient-centered care of older people that we identified from the literature [1, 4, 13, 36, 37] as described above: available evidence for older people, barriers to implementation of the CPG for older people, and tailoring treatment to older people context and preferences. Studies looking at Australian and Canadian CPGs suggest that only a few provide recommendations tailored to the special needs of older people [36, 37]. As far as we are aware, no studies have examined CPG recommendations for primary CVD prevention in older people.

## Methods

### Data sources

First, we searched two systematic reviews on CPGs for CVD prevention [23, 38] for CPGs discussing a combined, overall, global, total or absolute risk approach (from here on referred to as AR approach) and looked for updated version of these CPGs. Second, we performed our own systematic review to search for CPGs published after May 2009, the time-frame used in the previous studies, by searching the following three CPG-specific databases: the National CPG Clearinghouse (http://www.guideline.gov), G-I-N International CPG Library (http://www.g-i-n.net) and Trip database (https://www.tripdatabase.com). These databases are publicly available on the Internet. We conducted our search between April and December 2013.

### Inclusion/exclusion criteria

We included published CPGs on the assessment and/or management of CVD risk or two important risk factors for CVD: hypertension and high cholesterol. We only considered CPGs that mentioned an AR approach and specifically aimed to prevent a first CVD event (primary prevention). We excluded CPGs for diabetes, chronic kidney disease, HIV, lifestyle, general screening/prevention, pregnant or pediatric populations. We restricted the search to English language CPGs. Where more than one version of the same CPG was found we included only the most recent version.

### Search strategies

An information specialist was consulted to design the search strategy. The Medline search strategy consisted of two categories of subject headings (MESH) and title words (intersected by the Boolean term “AND”) covering (1) CVD, hypertension and dyslipidemia: (cardiovascular disease, heart disease, vascular disease, cardiac, coronary, ischemic, cardiovascular, cerebrovascular disorders, stroke, peripheral vascular diseases, renovascular or stroke, dyslipidemia, cholesterol, lipid, hypertension, blood pressure); and (2) clinical practice guidelines (practice guidelines, guidelines, clinical or practice and guidelines). We limited our search to English language articles. The Medline search syntax served as a basis for all search strategies. The full search strategy is available on request from the authors. We were also provided with CPGs through informal communication.

**Fig. 1:**
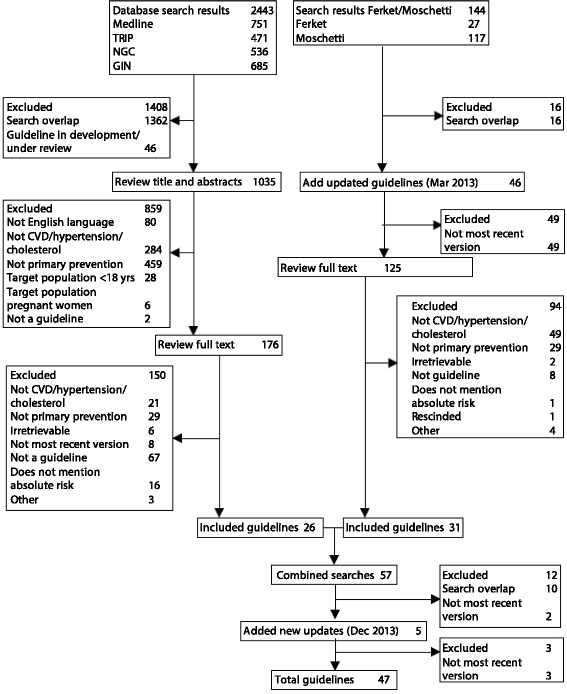
Summary of CPG search and review process

Two reviewers (JJ and SM) independently assessed all 1) titles and 2) abstracts/full text CPGs for potential eligibility, with discrepancies resolved by consensus if needed after discussion with all authors (see Fig. 1). For CPGs extracted from the two existing reviews, we combined step 1 and 2.

### Outcomes

When no specific age cut-off for older adults was indicated in the CPG, we looked for words that signified older age (e.g., senior, frail, elderly). *The primary outcome* was to identify the extent to which recommendations for CVD assessment/management in older adults (with or without comorbidities ) were given, more specifically any mention of: (1) available evidence for older people, (2) barriers to implementation of the CPG for older people, and (3) tailoring treatment to older people context and preferences. These criteria were selected from literature on the applicability of CPGs and patient centered care for older people [1, 4, 13, 36, 37]. See Table 1 for a more detailed description of these criteria.We only included statements that explicitly referred to older patients, which means that general statements for example  about the importance of patient involvement are not reported. Data were collected, summarized, and tabulated in an Excel spread sheet.

**Table 1:** Overview of criteria for CPG recommendations for older people*

Inclusion of information related to:		
1. Available evidence primary CVD prevention for older people	2. Barriers to implementation of the CPG for older people	3. Tailoring treatment to older people context and preferences
a. Evidence potential benefits/harms	a. Risk assessment complexity (e.g. measurement issues)	a. Patient preferences/values
b. Knowledge gaps	b. Risk management complexity (e.g. feasibility treatment targets)	b. Family preferences/values
	c. Time needed to treat to benefit in context of life expectancy	c. Patient context (e.g. quality of life, life expectancy, comorbidities)
	d. Meaningfulness outcomes for older people	d. Weighing benefits/harms
	e. Treatment adherence issues	e. Therapy prioritization
	f. Cognitive status	
	g. Social support/caregiver burden	

^*^Criteria were selected from literature on the applicability of CPGs and patient centered care for older people [1, 4, 13, 34, 35]

### Data collection and analysis

An initial data extraction plan was formulated (JJ), then discussed and revised with additional categories (JJ, CB, KM, SM), before all authors agreed on the approach. Three authors (JJ, SM, BN) independently extracted data from included CPGs and all CPGs were double coded to ensure accuracy and consistency. Any discrepancies were resolved by consensus to establish a single dataset. Data was collected on year of publication/update, principal disease, organization responsible for CPG development, country/region, whether it was an assessment CPG, a management CPG, or both. In line with the outcome measures, information was also extracted on any statements/recommendations that specifically addressed older people.

## Results

Of 1035 screened abstracts and 301 full text CPGs, 47 CPGs were included: 23 on assessment/management of CVD risk in general, 13 on cholesterol, and 11 on hypertension. Additional file 1: Appendix A summarizes the selected CPGs, including risk model and condition: CVD, hypertension or cholesterol. The denominator used is all CPGs (n = 47) unless statements specifically relate to hypertension or cholesterol (medication); in those instances the denominators are 34 (23 CVD + 11 hypertension CPGs) and 36 (23 CVD + 13 cholesterol CPGs), respectively.

### Inclusion of information related to older people

Almost all CPGs (92 %, 43/47) specified an age range for the target population and/or provided specific recommendations for older people. The statements provided ranged from general and brief to extensive and more specific recommendations providing references to relevant evidence, although the latter was rare (see Additional file 2: Appendix B). Thirty-eight percent of the CPGs (18/47) addressed older people with comorbidities, mostly just briefly mentioning that CVD risk management in older people should take into account comorbidities without specifying specific co-occurring conditions. In addition, 21 % (10/47) of the CPGs referred to frail older people, mostly recommending more caution with managing CVD risk in this group. Not in Table/Figure.

*“People aged 75 or older should be considered at increased risk of CVD (…). Assessment and treatment should be guided by the benefits and risks of treatment, informed preference and comorbidities that may make treatment inappropriate* [39]”.

“*Initial doses and subsequent dose titration should be more gradual because of a greater chance of undesirable effects, especially in very old and frail subjects* [40]”.

### Available evidence for older people

**Fig. 2:**
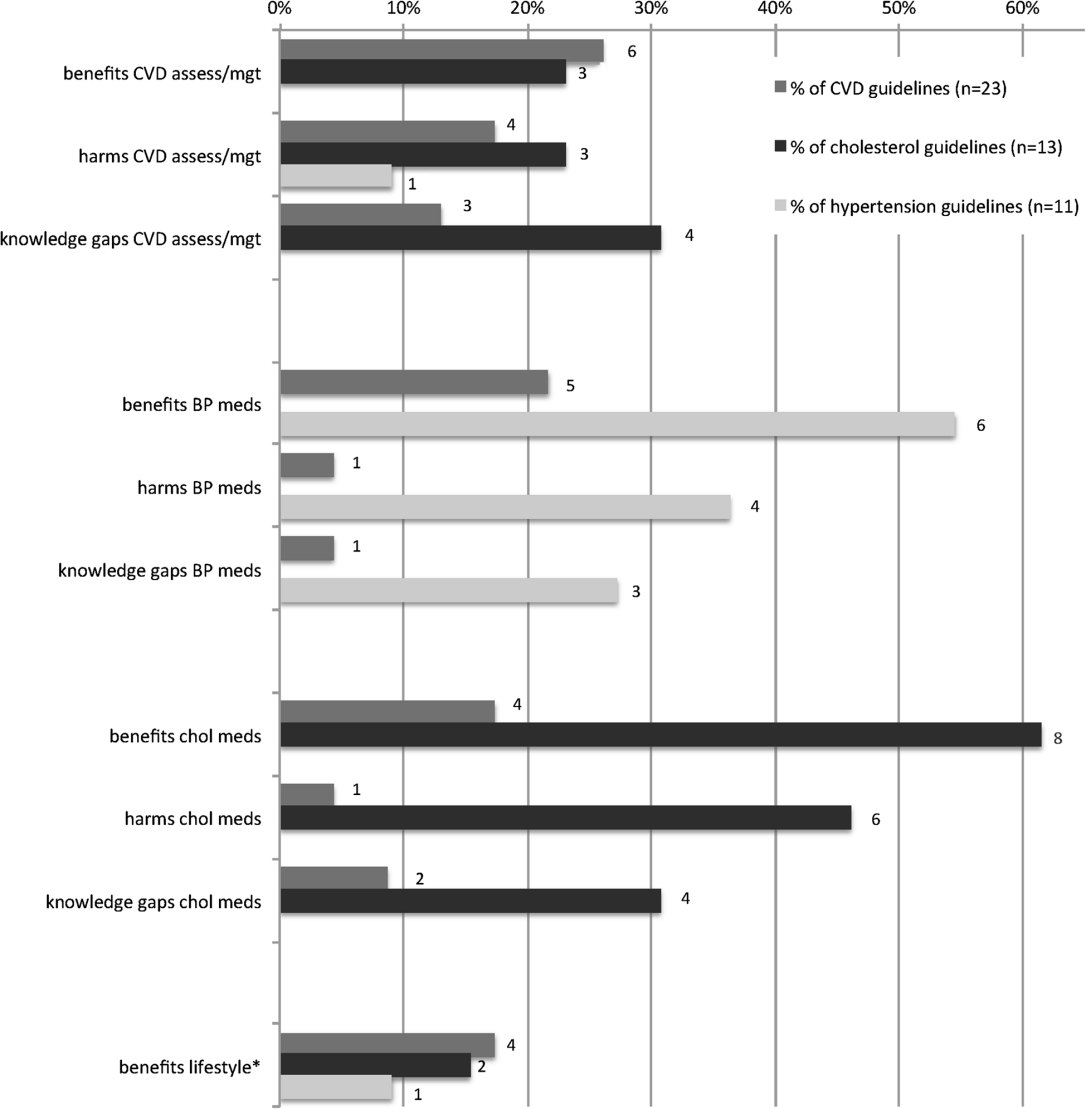
Available evidence different CVD risk management strategies (primary prevention) as mentioned in the CPGs (*n* = 47). Legend: *No harms or knowledge gaps mentioned for lifestyle. Abbreviations: assess/mgt = assessment/management, *BP* = blood pressure, *meds* = medication, chol = cholesterol

In total, 55 % (26/47) of the CPGs discussed the available evidence for primary CVD prevention in older people (see Fig. 2 for more detail). There was a disparity between discussion of potential benefits and harms, with benefits discussed more frequently (49 % of the CPGs, 23/47), than harms (38 % of the CPGs, 18/47). Knowledge gaps were discussed in 36 % of the CPGs (17/47). Additional file 2: Appendix B describes examples of brief and more extensive guideline recommendations for older people according to the three criteria for CPG recommendations in older people and Table 2 lists potential benefits and harms of different CVD risk management strategies (primary prevention) as mentioned in the CPGs.

**Table 2:** Potential benefits, harms and knowledge gaps of different CVD risk management strategies (primary prevention) for older people as mentioned in the CPGs (*n* = 47)

	Potential benefits	Potential harms	Knowledge gaps
**CVD risk assessment**	Provides an estimate of CVD* risk in older people	Risk models underestimate CVD risk for older people	Risk models not rigorously tested/reliable in older people
			Disagreement about the efficacy of risk assessment in older people (75+)
		Most CVD risk models focus on short term risk, and are therefore inevitably more likely to classify older people as at high risk and the young as at low risk	
	Beneficial in older patients with multiple risk factors and good quality of life		
	Repeated screening of cholesterol is less important as lipid levels are less likely to increase after age 65		
		Older people could be considered at high CVD risk based on their age while other risk factors are relatively low	
		Disease labeling healthy older people	
**CVD risk management overall**	CVD risk reduction	Risk of adverse effects is higher in older people	Limited available evidence for older people esp. older people with comorbidities and ‘oldest of old’ (age definitions are variable)
	Part of lifetime approach to CVD prevention		
		Resources are likely to be concentrated on older people, who may not be able to benefit in their remaining life (time needed to treat to benefit)	
	Similar relative benefit but greater absolute benefit for older people due to higher pre-treatment risk		
			Lack of generalizability of RCTs^†^ to older people in the community
	Similar benefit in old people as in young people (when taking into account higher case fatality rates after a CVD event in older people and temporal discounting of life years gained)		
			Disagreement about the efficacy of risk management in older people (75+)
		Costs associated with inappropriate prescribing in older people	
			Implication of knowledge gaps is that patient preferences and potential harms must be taken into account more, not just treatment benefits
	Improved quality of life		
**Both BP and cholesterol medication**	Morbidity/mortality benefit in older people	Risk of adverse effects is higher in older people, esp. frail and very old; risk is acceptable as long as the patient is carefully monitored	Limited available evidence for older people esp. frail old and older people with comorbidities; age definitions are variable
	Choice of drug should not be age dependent and is less important than degree of BP/cholesterol reduction		
			Lack of generalizability of RCTs to older people in the community
	Benefit for different treatment threshold/dosages in older people provided		
	Benefits provided for specific drugs		
	Benefits provided for different older age groups, age definitions are variable		
**Blood pressure medication**	No upper age limit to benefit	Risk of diabetes onset with thiazide diuretics	Limited available evidence on the benefits/harms of lowering SBP^§^ below certain threshold in older people
	Pre-existing very high risk might set a ceiling effect to the benefits of treatment; incl. in older patients		
		Risk of postural hypotension especially with alpha blockers	
			Older people are under-represented in trials vs. incentive to recruit more elderly to get enough high risk patients and CVD events for adequate power
	Morbidity but not mortality benefit in very old patients		
	Reducing BP^‡^ has benefits for other conditions beyond CVD (cognitive decline, dementia)		Unknown whether certain medication classes are superior to others in preventing cognitive decline
**Cholesterol medication**	Stronger evidence for the benefits of cholesterol medication for secondary prevention than primary prevention in older people	Small increase in all-cause mortality in older people	Association between high cholesterol and mortality weaker in older people
		Higher risk muscle toxicity in older people	
		Frailty is an additional risk factor for myopathy	
	Benefit for older people with risk factors other than age	Increased risk of cancer in older people	
	Benefit continuing well tolerated medication vs. starting medication	Very small risk of new-onset diabetes in older people but does not outweigh benefit	
**Lifestyle**	Benefit of healthy diet, physical activity, smoking, moderate alcohol intake	Not discussed	Not discussed
	Benefits of physical activity in older people include mortality benefit, improved quality of life and CVD risk reduction.		
	Weight loss and reduction of salt intake lowers blood pressure		
**Aspirin**	Reduced risk of CVD events/myocardial infarctions but older people need to have higher baseline risk for benefits to outweigh harms	Risk of adverse effects increases with age in particular gastrointestinal bleeding and hemorrhagic strokes	Not discussed

*CVD: cardiovascular disease; ^†^RCT: randomized controlled trial; ^‡^BP: blood pressure; ^§^SBP: systolic blood pressure

#### Potential benefits

Nineteen percent (9/47) of the CPGs discussed overall benefits of CVD assessment and management in older people, mostly referring to a similar relative benefit but greater absolute benefit for older people due to higher pre-treatment risk. Benefits of medication (morbidity/mortality benefit, positive effects on cognition) were discussed in 32 % (11/34) of the CVD/hypertension CPGs and 33 % percent (12/36) of the CVD/cholesterol CPGs. Fifteen percent of all CPGs (7/47) discussed the benefits of lifestyle management on CVD related morbidity and mortality.

#### Potential harms

Overall harms of CVD risk assessment and management in older people were discussed in 17 % (8/47) of the CPGs, mainly addressing that absolute risk assessment may underestimate risk in older people, and the use of resources for older people who are less likely to benefit due to short lifespan. Nineteen percent (7/36) of CVD/cholesterol CPGs mentioned potential harms of cholesterol lowering medication (e.g. muscle toxicity, increased risk of cancer) whereas only 15 % (5/34) of CVD/hypertension CPGs mentioned potential harms of blood pressure medication, mostly referring to risk of hypotension. None of the CPGs mentioned potential harms of lifestyle change in older people.

#### Benefit harm trade-off

Generally, the trade-off between benefits and harms of medication is more complicated in older people. Twenty-five percent (13/47) of the CPGs explicitly referred to making this trade-off, with statements varying from very general statements to more extensive discussions in a few cases (see for example *New Zealand Primary Care Handbook 2012* in Additional file 2: Appendix B).

#### Knowledge gaps

Knowledge gaps related to CVD risk assessment and management in older people were discussed in 15 % (7/47) of the CPGs, describing that most CVD risk models and interventions have not been thoroughly tested in older people, and the lack of generalizability of randomized clinical trials to older people in the community. Seventeen percent of the CVD/cholesterol CPGs (6/36), and 12 % of the CVD/hypertension CPGs (4/34) addressed limited available evidence of treatment with preventative CVD medication for older people, especially the very old/more frail. None of the CPGs addressed knowledge gaps around lifestyle interventions for older people.

#### Aspirin in CVD CPGs

Despite the lack of evidence for the use of aspirin in primary prevention of CVD [41], 9 % (4/47) of the CPGs mentioned the potential benefits in terms of CVD morbidity/mortality and specifically reduction of myocardial infarctions. Most of these CPGs (6 %, 3/47) also addressed the increased risk of gastrointestinal bleeds and/or hemorrhagic strokes and suggested that older people need higher baseline risk for benefits to outweigh harms (not in Figure/Table).

### Barriers to implementation of the CPG for older people

The majority of CPGs addressed one or more barriers to application of the guideline in older people (83 %, 39/47). Details of the specific barriers or complexities that were mentioned are discussed below and Additional file 3: Appendix C provides illustrative quotes from the CPGs.

### Risk assessment complexity

#### Absolute CVD risk (AR) approach

**Fig. 3:**
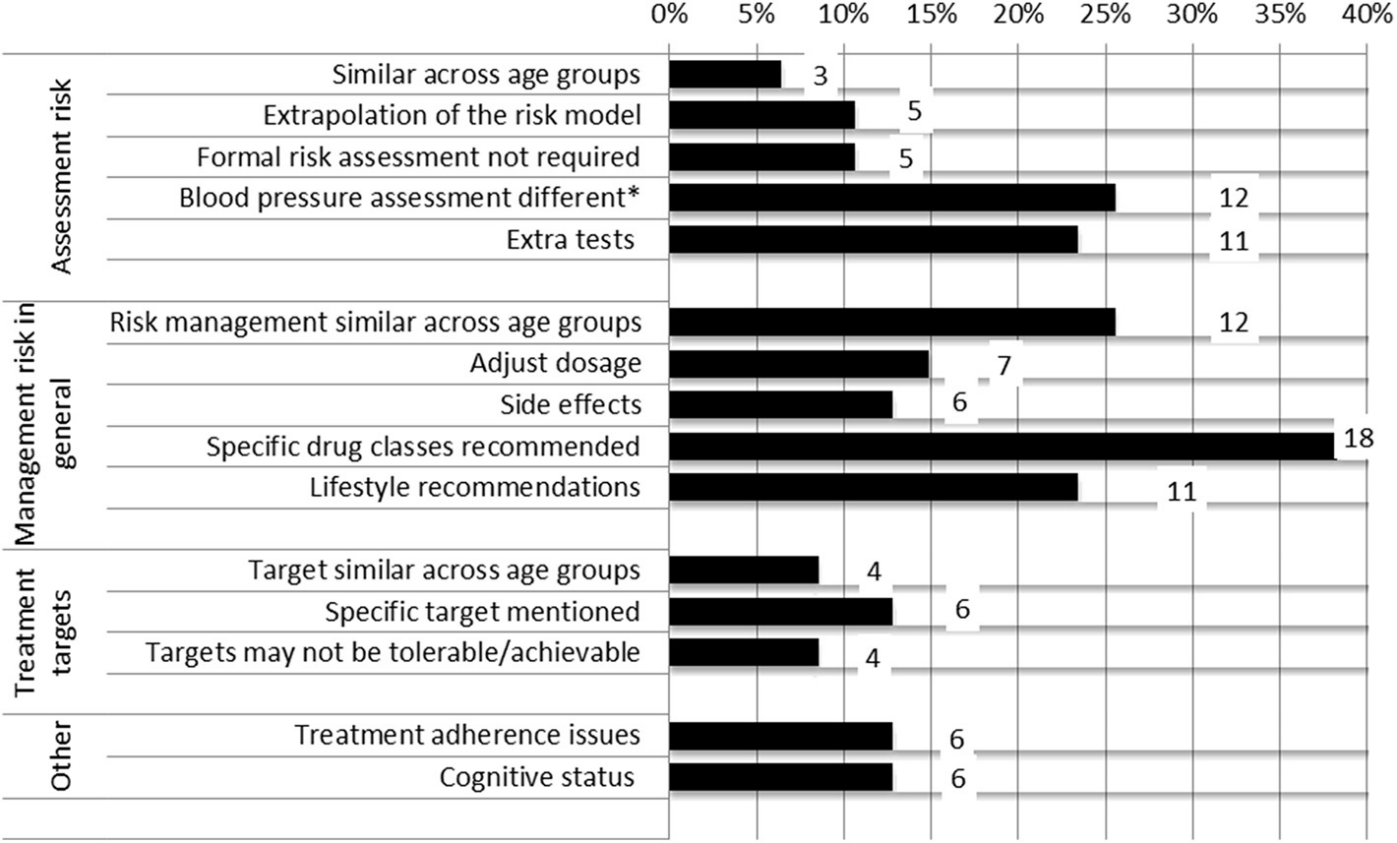
Barriers to implementation of the guideline for older people as mentioned in CPGs. Legend: Percentage of total number of guidelines *n* = 47; *Calculated out of 34 (23 CVD + 11 hypertension) CPGs

Forty-nine percent (23/47) of the CPGs addressed the complexities CVD risk assessment in older people (Fig. 3). For AR assessment, some CPGs (6 %, 3/47) recommended a similar approach in younger versus older people, whereas other CPGs (11 %, 5/47) advised to extrapolate the risk model for older people outside the target age range, either by using the upper age cut-off of the model or by providing adjusted risk scores for older people. Eleven percent of the CPGs (5/47) stated that older people above a certain age (usually > 80 years) or older people with additional risk factors (e.g. hypertension, large pulse pressure, smokers, diabetes) can be assumed to be at high risk without a formal assessment*.*

#### Blood pressure measurement

Thirty-five percent of the CVD/hypertension CPGs (12/34) addressed blood pressure measurement in older people, including recommendations to measure blood pressure both lying/sitting and standing to document postural hypotension, and information about the higher predictive value of systolic blood pressure (SBP) versus diastolic blood pressure (DBP) in determining CVD risk, especially in older people.

#### Additional tests

Twenty-three percent (11/47) of the CPGs mentioned the use of additional tests in the assessment and management of CVD risk in older people, including ankle-brachial index (ABI), ultrasound, wave velocity and echocardiography to detect possible arterial disease, cognitive tests and MRI to detect silent brain infarcts and creatine kinase tests before initiating treatment with cholesterol medication. This is despite the lack of good evidence for the use of tests above the Framingham risk equation.

### Risk management complexity

#### Management in general

Sixty-six percent (31/47) of the CPGs addressed management of CVD risk in older people. Twenty-six percent (12/46) stated that primary CVD prevention is generally similar for older versus younger people. However, age-specific recommendations were also provided, often by the same CPGs. Most commonly, CPGs provided a list of suitable and unsuitable pharmacotherapy (38 %, 18/47) and lifestyle strategies (23 %, 11/47) for older people. Thirteen percent (6/47) addressed side effects that are more likely in older people, most notably increased risk of muscle toxicity with cholesterol lowering medication. Common recommendations for older people were to closely monitor side effects and adjust medication dosage (15 %, 7/47) if need be.

#### Treatment targets

Treatment targets for older people were addressed in 19 % (9/47) of the CPGs. Some CPGs recommended similar targets for younger and older people (9 %, 4/47) whereas other CPGs commented that targets may be difficult to achieve (9 %, 4/47) or recommended specific, less stringent targets for older people (13 %, 6/47). For example, one CPG recommended a target blood pressure below 150/90 mmHg in people aged 80 years and over versus 140/90 mmHg in younger people [42]. None of the cholesterol CPGs discussed treatment targets for older people.

#### Cognition and treatment adherence

Cognition in older people was addressed in in 13 % (6/47) of CPGs, describing the relation between hypertension/high cholesterol and cognitive impairment (see applicability of evidence) or the relation between cognitive function and adherence. Thirteen percent of the CPGs (6/47) mentioned that treatment adherence is generally lower in older people, especially in those with comorbidities and cognitive/functional impairments. One CPG recommended to improve adherence by simplifying the medication regimen. None of the guidelines discussed social support or caregiver burden.

### Tailoring treatment to older people context and preferences

#### Patient context and preferences

**Fig. 4:**
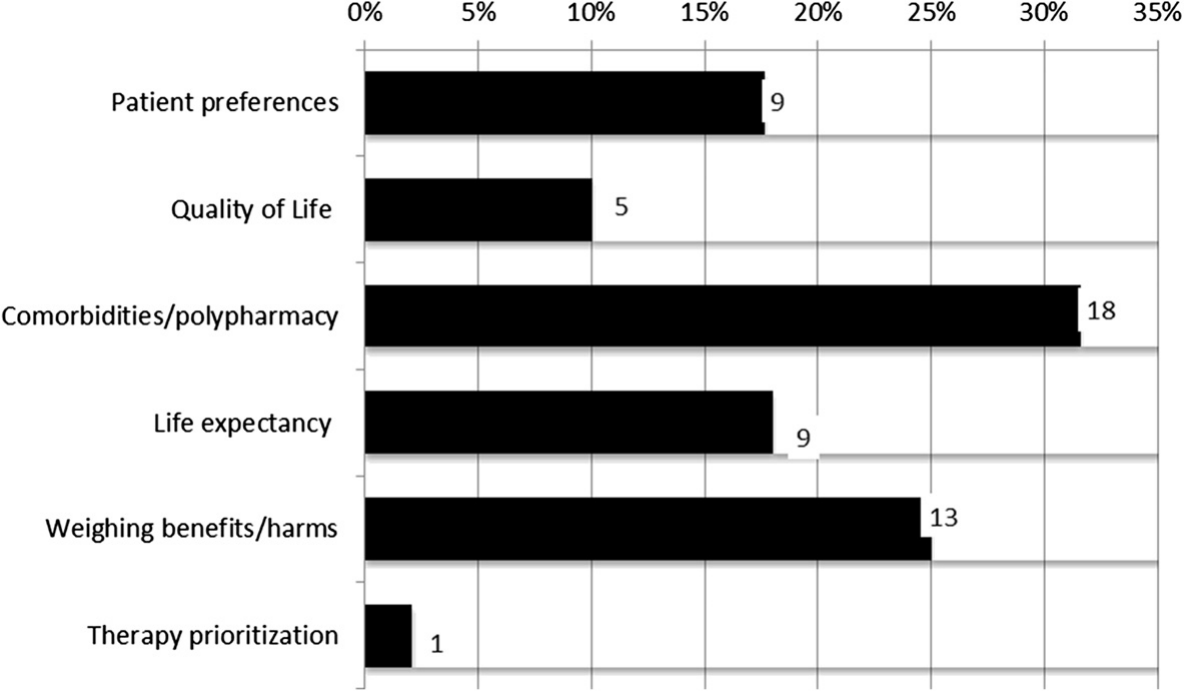
Tailoring treatment to older people context and preferences as mentioned in CPGs. Legend: Percentage of total number of guidelines *n* = 47

More than half of the CPGs (58 %, 27/47) addressed older patient context to some extent. Most commonly (32 %, 15/47) CPGs recommended that the decision to assess and/or manage CVD risk in older people should be based on clinical judgment taking into account comorbidities/polypharmacy (38 %, 18/47), patient preferences (19 %, 9/47), life expectancy and/or time needed to treat to benefit (19 %, 9/47), and quality of life (11 %, 5/47). These factors were often presented in list form, instructing clinicians to apply the CPGs with consideration of individual patient context but offering no specific guidance on how to do this. None of the CPGs addressed preferences of family members/companions (Fig. 4).

#### Weighing benefits/harms and therapy prioritization

Twenty-eight percent (13/47) of the CPGs mentioned weighing up the benefits and harms of CVD prevention for older people (see Table 2) and only one CPG explicitly mentioned the importance of prioritizing treatment in older people, albeit as a side issue.

#### Deprescribing

Discontinuation of medication in older people was not considered in any of the CPGs, although one CPG explicitly mentioned that “*(…) there is no reason for interrupting a successful and well tolerated therapy when a patient reaches* 80 years of age [40].”

## Discussion

Ninety-two percent of the included CPGs (43/47) referred to older adults to some extent, but the specific issues important in deciding about primary cardiovascular disease (CVD) prevention in older patients were mostly not adequately addressed. There was very limited discussion of frail older people and older people with comorbidities, a group for whom management is particularly challenging due to potential drug-drug and disease-drug interactions and competing health priorities [10, 43]. Only 55 % of the CPGs discussed available evidence for primary CVD prevention in older people and knowledge gaps. Potential benefits (in terms of morbidity, mortality and improved cognition) were discussed more extensively than harms (e.g. risk of hypotension with blood pressure medication), especially for hypertension medication and lifestyle recommendations. This is an important finding as even though older peoples’ preferences to take medication for primary CVD prevention vary widely [15] they are relatively insensitive to its benefit but highly sensitive to its adverse effects [14, 44], suggesting that clinical CPGs need to place emphasis on both benefits and harms, especially for older people. More specific adverse effects were mentioned for cholesterol medication than blood pressure medication, for example only a few CPGs mentioned an increased risk of fall injuries as a result of blood pressure medication in older people [31]. Evidence for lifestyle management in older adults was brief in most CPGs, with generally no information provided on the specific benefits or effects on CVD or other outcomes for older people, the amount of lifestyle change needed to benefit, or differences between age groups. None of the guidelines made recommendations about when treatment could or should be stopped. Use of medication to prevent CVD in older people should be carefully monitored as the benefit-harm trade-off may change if people, for example, become increasingly frail [45].

Although the majority of the included CPGs provided one or more statements describing under which circumstances the CPG might be difficult to implement for older people, addressing issues such as lack of validation of the risk models, increased risk side of effects, and the need to adjust treatment targets, recommendations are varied and brief. Given the heterogeneity of the older population, and widely varying treatment (outcome) preferences, treatment decision making needs to be tailored to the specific circumstances of the individual older adult, taking into account their preferences and values [4, 20]. Half of the CPGs recommended clinicians to apply the CPGs with consideration of individual older patient context (e.g. comorbidities, life expectancy), but did not offer specific guidance. Even fewer CPGs mentioned the importance of older patient involvement in decision making and none of the CPGs mentioned the importance of family members/companions.

### How our findings compare with other CPG assessments

Consistent with our findings, reviews of Canadian [37] and Australian [36] CPGs on prevalent chronic conditions in the older population found that although most CPGs refer to the older population to some extent, only a handful of them adequately address issues related to older patients, especially patients with comorbidities. One recent review of CPGs for patients with type-2 diabetes mellitus showed that the impact of multiple comorbidities, patient’s socio personal contexts, and patients’ personal values and preferences were only narrowly addressed in most CPGs [46]. Although this review did not focus on older people specifically, it concurs with the limited attention to older patient context and patient involvement the CPGs included in the current study, and suggests it is an important issue with CPGs more broadly.

### Limitations and strengths

The main limitation of our work is that we only looked at statements specifically referring to older people and it therefore does not allow for a comparison between recommendations for older versus younger adults. It also means that we did not include statements related to comorbidities or frailty that may have been applicable to older people, if they did not specifically address this group. However, we know from previous studies that CPGs rarely consider patients with comorbidities [36, 37, 46] so it is unlikely that including more general statements would have changed our results. Moreover, from the viewpoint of a clinician having to decide about the management of an older patient, guidelines would need to be clearly signposted as relevant for older people rather than requiring extraction from general text about management. Since our main objective was to examine the extent to which CVD CPGs address older adults, we did not carry out a formal quality evaluation of the CPGs. We limited our eligibility criteria to CPGs published in English.

Finally, we did not include the recently published JBS3 [47], NICE CG181 [48] and NHLBI JNC8 CPG [49] in the review as these were published after our last search date (31 December 2013). However, these CPGs appear to have a similar pattern to our main findings, with the exception of NICE CG181. In both the JBS3 and JNC8 guidelines, the discussion of older people is mainly limited to a recommendation to adjust treatment targets and/or thresholds The JBS8 has added an adjusted treatment threshold for people aged ≥80 years (BP <150/90 mm Hg, or <150/85 mm Hg if ambulatory or home BP monitoring is used). JNC8 now recommends an adjusted treatment threshold and target for people aged ≥60 years (SBP of 150 mmHg or DBP of 90 mmHg). Interestingly, the section on hypertension in older people in JNC7 guideline was removed in the JNC8 update. The updated NICE CG181 guideline provides much more extensive discussion of CVD risk management in older people than the previous version (CG67) [39]. In CG67 the main recommendation for older people had been to use clinical judgement to assess risk in people aged 75 or older but that this group could be considered at increased risk of CVD and is likely to benefit from statin treatment. In CG181 the major change is to explicitly recommend using the QRISK2 risk assessment, which has been validated in people up to, and including age 84 years. People aged 85 or older are considered to be at increased risk of CVD because of age alone and the CPG recommends considering statin treatment in this group. However, detail is added to make it explicit that there is limited evidence in older age groups, that the benefit may only be in reduced non-fatal MI and that consideration of risk and benefits and factors such as polypharmacy, comorbidity, frailty and life expectancy and informed patient preference are particularly important. It is also pointed out that there is a need for more research on the effectiveness of statin therapy in older people. The main strengths of our work includes the systematic approach, the use of a literature based data extraction method and the rigor of our analysis by employing double coding of CPG selection and data extraction.

### Implications for CPG development and research

Clinical practice guidelines set out a template for best practice for patient management to guide and support clinicians in their care of patients. It is clear from our review that even though clinicians report wanting additional advice about caring for older patients, the current CPGs fail to provide adequate guidance in the vast majority of cases. There is a clear need for randomized trials on primary CVD prevention in older people (with inclusion of the frail older person with comorbidities) measuring outcomes that are meaningful to older people, to enable evidence based decision making for this group. At the same time, our results show that the majority of the CPGs do not even report the evidence that is available [27, 28, 31–34], rendering it almost impossible for older patients and their clinicians to make an informed decision about whether and how to manage CVD risk. CPG developers should think about ways to present available evidence for older people in the CPGs or at least to highlight the uncertainty around the evidence for this group. CPGs should be structured so that they may be easily adapted to older people with comorbidities, clearly present potential benefits and harms to allow for comparison, and are conducive to shared decision making. This may require the use of new technology [50]. Existing guidance on risk communication [51] (although mainly limited to younger people), use of prognostic indices [12] and health outcome prioritization tools [13, 15] for decision making in older people could help inform this process.

## Conclusion

This study shows that only few primary CVD prevention CPGs adequately address important issues common in the care of older people. Given the changing demographics and aging population worldwide, this is of major significance. Future CPGs should provide more detailed evidence and guidance on the management of older people with multiple comorbidities and frailty, to ensure that discussions about the risk and benefits (and uncertainties) of different management options can take place and clinician and patients can make a shared decision that is tailored to the individual patient’s circumstances and preferences. CPGs must be designed to provide the best possible advice and support to clinicians for the care of their patients. Care of older patients will not improve until important aspects of their care are addressed by health care providers. Achieving high quality guidelines to support clinicians is an essential part of this process.

## Additional files

Additional file 1: Appendix A. Overview CPG recommendations older people (n=47), organised by medical condition then by year of publication (reverse chronological) (PDF 354 kb) Additional file 2: Appendix B. Examples of brief and more extensive guideline recommendations for older people according to the three criteria for CPG recommendations older people. (PDF 525 kb) Additional file 3: Appendix C. Supporting Quotes from the CPGs (PDF 436 kb)

## Abbreviations

AR: Absolute risk
CPG: Clinical Practice Guideline
CVD: Cardiovascular Disease
DBP: Diastolic blood pressure
SBP: Systolic blood pressure
