# Supplementary material for: Systematic review of clinical practice guidelines recommendations about primary cardiovascular disease prevention for older adults
Source: BMC Fam Pract. 2015 Aug 20;16:104. doi: 10.1186/s12875-015-0310-1 (PMC4546022; doi:10.1186/s12875-015-0310-1)
Supplement: Additional file 1: Appendix A. — Overview CPG recommendations older people (n=47), organised by medical condition then by year of publication (reverse chronological) (PDF 354 kb) [file 12875_2015_310_MOESM1_ESM.pdf]

Appendix A. Overview CPG recommendations older people (n=47), organised by medical condition then by year of publication (reverse chronological)

Medical Condition	Organisation / Guideline Developer	Year of publication/ update	Country/region	Main risk model used
CVD	British Joint Societies [1]	2005	UK	Joint British Societies Risk Prediction Chart
CVD	World Health Organization [2]	2007	International	Various risk models mentioned
CVD	Scottish Intercollegiate Guidelines Network [3]	2007	Scotland	ASSIGN
CVD	European Society of Cardiology [4]	2007	Europe	SCORE
CVD	British Colombia Medical Association/Ministry of Health [5]	2008	Canada	UKPDS and Framingham
CVD	The Endocrine Society [6]	2008	International	Framingham, PROCAM, SCORE
CVD	National Institute for Health and Clinical Excellence [7]	2008	UK	Various risk models mentioned
CVD	Ministry of Health, Malaysia [8]	2008	Malaysia	Various risk models mentioned
CVD	US Preventive Services Task Force [9]	2009	US	Framingham
CVD	National Academy of Clinical Biochemistry [10]	2009	US	Framingham
CVD	Toward Optimized Practice Program, Alberta [11]	2009	Canada	Framingham
CVD	Second Indo-US Health Summit Expert Panel [12]	2009	US/India	Various risk models mentioned
CVD	American College of Cardiology Foundation and American Heart Association [13]	2010	US	Various risk models mentioned
CVD	American College of Preventive Medicine [14]	2011	US	Framingham
CVD	Ministry of Health Clinical Practice Guidelines Workgroup [15]	2011	Singapore	Framingham adapted to Singapore population
CVD	American Heart Association [16]	2011	US	Framingham or Reynolds Risk Score
CVD	European Society of Cardiology [17]	2012	Europe	Framingham or Score
CVD	National Vascular Disease Prevention Alliance, Australia [18]	2012	Australia	Framingham
CVD	National Screening Committee [19]	2012	UK	Various risk models mentioned
CVD	New Zealand Guidelines Group [20]	2012	New Zealand	New Zealand CVD Risk Charts
CVD	Map of Medicine - NHS choices [21]	2013	UK	Various risk models mentioned
CVD	Cardiovascular Disease Risk Assessment Steering Committee [22]	2013	New Zealand	New Zealand CVD Risk Charts
CVD	American College of Cardiology/American Heart	2013	US	New Pooled Cohort ASCVD Risk Equations

	Association [23]			
Dyslipidemia	United States Department of Veterans Affairs / Department of Defense [24]	2006	US	Framingham
Dyslipidemia	US Preventive Services Task Force [25]	2008	US	Various risk models mentioned
Dyslipidemia	Caribbean Cardiac Society [26]	2008	Caribbean	Risk model not specified
Dyslipidemia	University of Michigan Health System [27]	2009	US	Framingham
Dyslipidemia	Ontario Association of Medical Laboratories [28]	2010	Canada	Framingham
Dyslipidemia	European Society of Cardiology & European Artherosclerosis Society [29]	2011	Europe	SCORE (but other risk models mentioned)
Dyslipidemia	Institute for Clinical Systems Improvement [30]	2011	US	Framingham
Dyslipidemia	Ministry of Health, Malaysia [31]	2011	Malaysia	Framingham (but other risk models mentioned)
Dyslipidemia	Canadian Cardiovascular Society [32]	2013	Canada	Framingham
Dyslipidemia	American Association of Clinical Endocrinologists [33]	2012	US	Framingham or Reynolds Risk Score
Dyslipidemia	South African Heart Association/Lipid and Atherosclerosis Society of Southern Africa [34]	2012	South Africa	Framingham
Dyslipidemia	American College of Cardiology/American Heart Association [35]	2013	US	New Pooled Cohort ASCVD Risk Equations
Dyslipidemia	Michigan Quality Improvement Consortium [36]	2013	US	Framingham
Hypertension	Heart and Stroke Foundation of Ontario, Registered Nurses' Association of Ontario [37]	2005/2009*	Canada	Various risk models mentioned
Hypertension	United States Department of Veterans Affairs / Department of Defense [38]	2004	US	Risk model not specified
Hypertension	National Heart, Lung, and Blood Institute [39]	2004	US	Framingham
Hypertension	European Society of Cardiology & European Society of Hypertension [40]	2007	Europe	Heart-Score based on SCORE
Hypertension	US Preventive Services Task Force [41]	2007	US	Risk model not specified
Hypertension	Latin American Expert Group [42]	2009	Latin America	Own risk charts
Hypertension	National Heart Foundation of Australia [43]	2010	Australia	Australian Risk Charts
Hypertension	National Institute for Health and Clinical Excellence [44]	2011	UK	Various risk models mentioned
Hypertension	South African Hypertension Society [45]	2011	South Africa	Risk chart based on ESH/ESC guidelines
Hypertension	Institute for Clinical Systems Improvement [46]	2012	US	Framingham
Hypertension	Canadian Hypertension Education Program [47]	2013	Canada	Risk model not specified

CVD = cardiovascular disease, * 2009 is update of 2005

REFERENCES

1. British Cardiac Society, British Hypertension Society, Diabetes UK, HEART UK, Primary Care Cardiovascular Society, The Stroke Association. JBS 2: Joint British Societies' guidelines on prevention of cardiovascular disease in clinical practice. *Heart*. 2005;91:v1-v52.
2. WHO. Prevention of Cardiovascular Disease: Guidelines for assessment and management of cardiovascular risk. WHO, Geneva. 2007.
3. Scottish Intercollegiate Guidelines Network. Risk estimation and the prevention of cardiovascular disease: A national clinical guideline. NHS, Edinburgh. 2007.
4. Collins P, Rosano G, Casey C, Daly C, Gambacciani M, Hadji P, Kaaja R, Mikkola T, Palacios S, Preston R, Simon T, Stevenson J, Stramba-Badiale M. Management of cardiovascular risk in the peri-menopausal woman: a consensus statement of European cardiologists and gynaecologists. *Eur Heart J*. 2007;28:2028-40.
5. Guidelines and Protocols Advisory Committee. Cardiovascular Disease - Primary Prevention. British Colombia Medical Association, British Colombia Ministry of Health Service, British Colombia. 2008.
6. Rosenzweig JL, Ferrannini E, Grundy SM, Haffner SM, Heine RJ, Horton ES, Kawamori R. Primary prevention of cardiovascular disease and type 2 diabetes in patients at metabolic risk: an Endocrine Society clinical practice guideline. *J Clin Endocrinol Metab*. 2008;93:3671-89.
7. National Institute for Health and Clinical Excellence. NICE Clinical Guideline 67: Lipid modification - Cardiovascular risk assessment and the modification of blood lipids for the primary and secondary prevention of cardiovascular disease. National Institute for Health and Clinical Excellence , London. 2010.
8. Ministry of Health Malaysia, Academy of Medicine Malaysia, National Heart Association of Malaysia. Prevention of Cardiovascular Disease in Women (1st edition). Ministry of Health, Putrajaya. 2008.
9. U.S. Preventive Services Task Force. Aspirin for the prevention of cardiovascular disease: U.S. Preventive Services Task Force Recommendation Statement. *Ann Intern Med*. 2009;150:396-404.
10. Myers, GL (ed), National Academy of Clinical Biochemistry. Laboratory Medicine Practice Guidelines - Emerging Biomarkers for Primary Prevention of Cardiovascular Disease and Stroke. American Association for Clinical Chemistry; Washington, DC. 2009.
11. Towards Optimized Practice Program. Guideline for Management of Modifiable Risk Factors in Adults at High Risk for Cardiovascular Events. Towards Optimized Practice Program, Edmonton. 2009.
12. Second Indo-US Health Summit Expert Panel. Recommendations of the second Indo-U.S. health summit on prevention and control of cardiovascular disease among Asian Indians. *Indian Heart Journal*. 2009;61:265-74.
13. Greenland P, Alpert JS, Beller GA, Benjamin EJ, Budoff MJ, Fayad ZA, Foster E, Hlatky MA, Hodgson JM, Kushner FG, Lauer MS, Shaw LJ, Smith SC, Taylor AJ, Weintraub WS, Wenger NK. 2010 ACCF/AHA guideline for assessment of cardiovascular risk in asymptomatic adults. A report of the American College of Cardiology Foundation/American Heart Association Task Force on Practice Guidelines. *Journal of the American College of Cardiology*. 2010;56(25):e50
14. Lim LS, Haq N, Mahmood S, Hoeksema L, ACPM Prevention Practice Committee. Atherosclerotic Cardiovascular Disease Screening in Adults American College of Preventive Medicine Position Statement on Preventive Practice. *Am J Prev Med*. 2011;40(3):380

15. Goh LG, Chua T, Kang V, Kwong KH, Lim WY, Low LP, Pereira J, Venketasubramanian N, Sethi SK, Sum CF, Tan CL, Tan HK, Tan SM, Wong TK. Ministry of Health Clinical Practice Guidelines: screening of cardiovascular disease and risk factors. *Singapore Med J*. 2011;52:220-5.
16. Mosca L, Benjamin EJ, Berra K, Bezanson JL, Dolor RJ, Lloyd-Jones DM, Newby LK, Pina IL, Roger VL, Shaw LJ, Zhao D, Beckie TM, Bushnell C, D'Armiento J, Kris-Etherton PM, Fang J, Ganiats TG, Gomes AS, Gracia CR, Haan CK, Jackson EA, Judelson DR, Kelepouris E, Lavie CJ, Moore A, Nussmeier NA, Ofili E, Oparil S, Ouyang P, Pinn VW, Sherif K, Smith SC, Jr., Sopko G, Chandra-Strobos N, Urbina EM, Vaccarino V, Wenger NK. Effectiveness-based guidelines for the prevention of cardiovascular disease in women--2011 update: a guideline from the American Heart Association. *Circulation*. 2011;123:1243-62.
17. Perk J, De Backer G, Gohlke H, Graham I, Reiner Z, Verschuren M, Albus C, Benlian P, Boysen G, Cifkova R, Deaton C, Ebrahim S, Fisher M, Germano G, Hobbs R, Hoes A, Karadeniz S, Mezzani A, Prescott E, Ryden L, Scherer M, Syvanne M, Scholte op Reimer WJ, Vrints C, Wood D, Zamorano JL, Zannad F, European Association for Cardiovascular Prevention, Rehabilitation and Guidelines, ESC Committee for Practice Guidelines. European Guidelines on cardiovascular disease prevention in clinical practice (version 2012). The Fifth Joint Task Force of the European Society of Cardiology and Other Societies on Cardiovascular Disease Prevention in Clinical Practice. *Eur Heart J*. 2012;33:1635-701.
18. National Vascular Disease Prevention Alliance. Guidelines for the management of absolute cardiovascular disease risk. NVDPA, Australia. 2012.
19. National Screening Commitee. The Handbook for Vascular Risk Assessment, Risk Reduction and Risk Management. University of Leicester, Leicester. 2012.
20. New Zealand Guideline Groups. New Zealand Primary Care Handbook 2012. 3rd edition. NZGG, Wellington. 2012.
21. Map of Medicine. Cardiovascular disease (CVD) risk assessment and primary prevention. NHS, London. 2013.
22. Cardiovascular Disease Risk Assessment Steering Group, New Zealand Guidelines Group. New Zealand Primary Care Handbook 2012 (updated 2013): Cardiovascular Disease Risk Assessment. New Zealand Guidelines Group, Wellington. 2013.
23. Goff DC, Lloyd-Jones DM, Bennett G, Coady S, D'Agostino RB, Gibbons R, Greenland P, Lackland DT, Levy D, O'Donnell CJ, Robinson J, Schwartz JS, Shero ST, Smith SC, Sorlie P, Stone NJ, Wilson PW. ACC/AHA Guideline on the Assessment of Cardiovascular Risk: A Report of the American College of Cardiology/American Heart Association Task Force on Practice Guidelines. *Circulation*. 2013.
24. VA/DoD, The Management of Dyslipidemia Working Group. VA/DoD Clinical Practice Guideline for the Management of Dyslipidemia. Department of Veterans Affairs; Washington, DC. 2006.
25. U.S. Preventive Services Task Force. Screening for lipid disorders in adults: U.S. Preventive Services Task Force recommendation statement. Agency for Healthcare Research and Quality; Rockville, MD. 2008.
26. Chung EE and Caribbean Cardiac Society. Best Practice Guidelines for Hypercholesterolemia. 2008.
27. University of Michigan Health System. Screening and Management of Lipids. University of Michigan. 2009.
28. Ontario Association of Medical Laboratories. Guideline for Lipid Testing in Adults (CLP017). Ontario Association of Medical Laboratories, North York. 2010.
29. The Task Force for the management of dyslipidaemias of the European Society of Cardiology (ESC) and the European Atherosclerosis Society (EAS). ESC/EAS Guidelines for the management of dyslipidaemias. *Eur Heart J*. 2011;32:1769-818.
30. Woolley T, Kopecky S, O'Connor P, Susan Hanson, Dupras D, Kofron P, Krueger K, Needham R, Johnson DJ, Mikel K. Bofenkamp, Groen S, Institute for Clinical Systems Improvement. Healthcare Guideline: Lipid Management in Adults. Institute for Clinical Systems Improvement, Bloomington. 2011.

31. Ministry of Health, National Heart Association of Malaysia, Academy of Medicine. 4th Edition of Clinical Practice Guidelines: Management of Dyslipidemia 2011. Ministry of Health, Putrajaya. 2011.
32. Anderson TJ, Gregoire J, Hegele RA, Couture P, Mancini GB, McPherson R, Francis GA, Poirier P, Lau DC, Grover S, Genest J, Jr., Carpentier AC, Dufour R, Gupta M, Ward R, Leiter LA, Lonn E, Ng DS, Pearson GJ, Yates GM, Stone JA, Ur E. 2012 update of the Canadian Cardiovascular Society guidelines for the diagnosis and treatment of dyslipidemia for the prevention of cardiovascular disease in the adult. *The Canadian Journal of Cardiology*. 2013;29:151-67.
33. Jellinger PS, Smith DA, Mehta AE, Ganda O, Handelsman Y, Rodbard HW, Shepherd MD, Seibel JA and AACE Taskforce for Management of Dyslipidemia and Prevention of Atherosclerosis. American Association of Clinical Endocrinologists' Guidelines for Management of Dyslipidemia and Prevention of Artherosclerosis. *Endocrine Practice*. 2012;18(Suppl 1)
34. Klug E. South African Dyslipidaemia Guideline Consensus Statement. *South African Medical Journal*. 2012;102:178-188.
35. Stone NJ, Robinson J, Lichtenstein AH, Barey Mertz CN, Blum CB, Eckel RH, Goldberg AC, Gordon D, Levy D, Lloyd-Jones DM, McBride P, Schwartz JS, Shero ST, Smith SC, Watson K, Wilson PW. ACC/AHA Guideline on the Treatment of Blood Cholesterol to Reduce Atherosclerotic Cardiovascular Risk in Adults: A Report of the American College of Cardiology/American Heart Association Task Force on Practice Guidelines. *Circulation*. 2013.
36. Michigan Quality Improvement Consortium. Screening and Management of Hypercholesterolemia.Michigan Quality Improvement Consortium, Southfield (MI). 2013.
37. Registered Nurses Association of Ontario. Nursing Management of Hypertension. 2005. Supplement: 2009. Heart and Stroke Foundation of Ontario, Ontario. 2005.
38. VA/DoD, Management of Hypertension in the Primary Care Setting Working Group. VA/DoD Clinical Practice Guideline for Diagnosis and Management of Hypertension in the Primary Care Setting. Department of Veterans Affairs; Washinngton, DC. 2004.
39. U.S. Department of Health and Human Services, National Institutes of Health, National Heart, Lung, and Blood Institute, National High Blood Pressure Education Program. The Seventh Report of the Joint National Committee on Prevention, Detection, Evaluation, and Treatment of High Blood Pressure. U.S. Department of Health and Human Services. 2004.
40. The Task Force for the Management of Arterial Hypertension of the European Society for Hypertension (ESH) and of the European Society of Cardiology (ESC). 2007 Guidelines for the management of arterial hypertension. *Eur Heart J*. 2007;28:1462-536.
41. U.S. Preventive Services Task Force. Screening for high blood pressure: U.S. Preventive Services Task Force reaffirmation recommendation statement. *Annals of Internal Medicine*. 2007;147:783-786.
42. Sanchez RA, Ayala M, Baglivo H, Velazquez C, Burlando G, Kohlmann O, Jimenez J, Jaramillo PL, Brandao A, Valdes G, Alcocer L, Bendersky M, Ramirez AJ, Zanchetti A, Latin American Expert Group. Latin American guidelines on hypertension. *Journal of Hypertension*. 2009;27:905-922.
43. National Heart Foundation of Australia. Guide to management of hypertension 2008. Updated December 2010. National Heart Foundation of Australia. 2010.
44. National Institute for Health and Clinical Excellence. NICE Clinical Guideline 127: Hypertension. Clinical management of primary hypertension in adults. National Institute for Health and Clinical Excellence , London. 2011.
45. Seedat YK and Rayner BL. South African Hypertension Guideline 2011. *South African Medical Journal*. 2012;102:60-88.

46. Luehr D, Woolley T, Burke R, Dohmen F, Hayes R, Johnson M, Kerandi H, Margolis K, Marshall M, O'Connor P, Pereira C, Reddy G, Schlichte A, Schoenleber M and Institute for Clinical Systems Improvement. Health Care Guideline: Hypertension Diagnosis and Treatment. Institute for Clinical Systems Improvement, Bloomington. 2012.
47. Hackam DG, Quinn RR, Ravani P, Rabi DM, Dasgupta K, Daskalopoulou SS, Khan NA, Herman RJ, Bacon SL, Cloutier L, Dawes M, Rabkin SW, Gilbert RE, Ruzicka M, McKay DW, Campbell TS, Grover S, Honos G, Schiffrin EL, Bolli P, Wilson TW, Feldman RD, Lindsay P, Hill MD, Gelfer M, Burns KD, Vallee M, Prasad GV, Lebel M, McLean D, Arnold JM, Moe GW, Howlett JG, Boulanger JM, Larochelle P, Leiter LA, Jones C, Ogilvie RI, Woo V, Kaczorowski J, Trudeau L, Petrella RJ, Milot A, Stone JA, Drouin D, Lavoie KL, Lamarre-Cliche M, Godwin M, Tremblay G, Hamet P, Fodor G, Carruthers SG, Pylypchuk GB, Burgess E, Lewanczuk R, Dresser GK, Penner SB, Hegele RA, McFarlane PA, Sharma M, Reid DJ, Tobe SW, Poirier L, Padwal RS. The 2013 Canadian Hypertension Education Program recommendations for blood pressure measurement, diagnosis, assessment of risk, prevention, and treatment of hypertension. *The Canadian Journal of Cardiology*. 2013;29:528-42.
