# Supplementary material for: Systematic review of clinical practice guidelines recommendations about primary cardiovascular disease prevention for older adults
Source: BMC Fam Pract. 2015 Aug 20;16:104. doi: 10.1186/s12875-015-0310-1 (PMC4546022; doi:10.1186/s12875-015-0310-1)
Supplement: Additional file 2: Appendix B. — Examples of brief and more extensive guideline recommendations for older people according to the three criteria for CPG recommendations older people. (PDF 525 kb) [file 12875_2015_310_MOESM2_ESM.pdf]

Appendix B. Examples of brief and more extensive guideline recommendations for older people according to the three criteria for CPG recommendations older people.

**Guideline name**	4th Edition of Clinical Practice Guidelines: Management of Dyslipidemia 2011 [1]	2012 update of the Canadian Cardiovascular Society guidelines for the diagnosis and treatment of dyslipidemia for the prevention of cardiovascular disease in the adult [2]	New Zealand Primary Care Handbook 2012 (updated 2013): Cardiovascular Disease Risk Assessment [3]	2007 Guidelines for the management of arterial hypertension [4]
**Guideline developer**	Ministry of Health, Malaysia	Canadian Cardiovascular Society	New Zealand Guidelines Group	European Society of Cardiology
Available evidence	*Increasing age is a major risk factor for CVD and death. (…) The benefits of lipid lowering therapy for primary prevention in elderly individuals with no other risk factors besides dyslipidaemia are less well established*	*Practical tip. For patients older than 75 years of age, the Framingham model is not well validated.*	*Estimating risk: The evidence base to inform recommendations for lipid lowering in primary prevention in the elderly is limited. An assessment of the balance between the harms and benefits of treatment is more difficult in older than in younger people. Older people gain a similar relative benefit from cholesterol lowering, but are more likely to benefit in absolute terms (over the same time period) because of their much higher pre-treatment of cardiovascular risk.*	***Box 13 Antihypertensive treatment in the elderly***<br>- *Randomized trials in patients with systolic-diastolic or isolated systolic hypertension aged ≥ 60 years have shown that a marked reduction in cardiovascular morbidity and mortality can be achieved with antihypertensive treatment*<br>- *Drug treatment can be initiated with thiazide diuretics, calcium antagonists, angiotensin receptor antagonists, ACE inhibitors, and b-blockers, in line with general guidelines. Trials specifically addressing treatment of isolated systolic hypertension have shown the benefit of thiazides and calcium antagonists but subanalysis of other trials also shows efficacy of angiotensin receptor*

				antagonists<br>- *In subjects aged 80 years and over, evidence for benefits of antihypertensive treatment is as yet inconclusive. However, there is no reason for interrupting a successful and well tolerated therapy when a patient reaches 80 years of age.*
Barriers to implementation	*Global risk assessment using standard risk factors as mentioned earlier is generally less reliable in older persons*	*One approach is extrapolation of the modified FRS, and this approach identifies most subjects as having intermediate- to high-risk based on age*	*An assessment of the balance between the harms and benefits of treatment is more difficult in older than in younger people. Older people gain a similar relative benefit from cholesterol lowering, but are more likely to benefit in absolute terms (over the same time period) because of their much higher pre-treatment of cardiovascular risk. However, comorbidity is more common and the time available to derive benefit will be shorter.*	- *Initial doses and subsequent dose titration should be more gradual because of a greater chance of undesirable effects, especially in very old and frail subjects.*<br>- *BP goal is the same as in younger patients, i.e. < 140/90 mmHg or below, if tolerated. Many elderly patients need two or more drugs to control blood pressure and reductions to <140 mmHg systolic may be particularly difficult to obtain.*<br>- *Drug treatment should be tailored to the risk factors, target organ damage and associated cardiovascular and non-cardiovascular conditions that are frequent in the elderly. Because of the increased risk of postural hypotension, BP should always be measured also in the erect posture.*

Tailoring to older patient	*Clinical judgment and consideration of co-morbid factors, co-existing disease and functional age become essential in deciding the need for drug therapy in this situation*	*Though clinical studies are currently under way to address this group, at this point clinical judgment is required in consultation with the patient to determine the value of pharmacotherapy*	*However, comorbidity is more common and the time available to derive benefit will be shorter. Similarly, the patient's expectations should be taken into account in the shared decisions. Smoking cessation is beneficial at any age.*	*Drug treatment should be tailored to the risk factors, target organ damage and associated cardiovascular and non-cardiovascular conditions that are frequent in the elderly.*

REFERENCES

1. Ministry of Health, National Heart Association of Malaysia, Academy of Medicine. 4th Edition of Clinical Practice Guidelines: Management of Dyslipidemia 2011. Ministry of Health, Putrajaya. 2011.
2. Anderson TJ, Gregoire J, Hegele RA, Couture P, Mancini GB, McPherson R, Francis GA, Poirier P, Lau DC, Grover S, Genest J, Jr., Carpentier AC, Dufour R, Gupta M, Ward R, Leiter LA, Lonn E, Ng DS, Pearson GJ, Yates GM, Stone JA, Ur E. 2012 update of the Canadian Cardiovascular Society guidelines for the diagnosis and treatment of dyslipidemia for the prevention of cardiovascular disease in the adult. The Canadian Journal of Cardiology. 2013;29:151-67.
3. New Zealand Guidelines Group. New Zealand Primary Care Handbook 2012 (updated 2013): Cardiovascular Disease Risk Assessment. Ministry of Health, Wellington. 2013.
4. The Task Force for the Management of Arterial Hypertension of the European Society for Hypertension (ESH) and of the European Society of Cardiology (ESC). 2007 Guidelines for the management of arterial hypertension. Eur Heart J. 2007;28:1462-536.
