# Supplementary material for: Systematic review of clinical practice guidelines recommendations about primary cardiovascular disease prevention for older adults
Source: BMC Fam Pract. 2015 Aug 20;16:104. doi: 10.1186/s12875-015-0310-1 (PMC4546022; doi:10.1186/s12875-015-0310-1)
Supplement: Additional file 3: Appendix C. — Supporting Quotes from the CPGs (PDF 436 kb) [file 12875_2015_310_MOESM3_ESM.pdf]

Appendix C. Supporting Quotes

**Criteria for CPG recommendations for older people**	**Supporting Quotes**
Inclusion of information related to older people	*"People aged 75 or older should be considered at increased risk of CVD (…). Assessment and treatment should be guided by the benefits and risks of treatment, informed preference and comorbidities that may make treatment inappropriate." [1]*<br>*"Initial doses and subsequent dose titration should be more gradual because of a greater chance of undesirable effects, especially in very old and frail subjects." [2]*
**Barriers to implementation of the CPG for older people**	
Risk assessment complexity – absolute CVD risk approach	*"In adults aged over 74 (…) absolute cardiovascular risk over the next five years should be assessed using the Framingham Risk Equation. Calculation should be performed using the age of 74 years. Although the Framingham Risk Equation might underestimate risk in this population, available evidence suggests that this approach will provide an estimate of minimum cardiovascular risk." [3]*<br>*"Older patients with diabetes must be considered to be at high risk [of CVD]" [4]*<br>*"(…) Adults with any of the following conditions do not require absolute CVD risk assessment using the Framingham Risk Equation because they are already known to be at clinically determined high risk of CVD (EBR: Grade D): Diabetes and age >60 years (…)" [3]*
Risk assessment complexity – blood pressure measurement	*"SBP provides more appropriate classification and risk stratification than DBP in the elderly. (…) Pulse pressure (PP) (SBP–DBP) is only marginally stronger than SBP for risk stratification in individuals over age 60, but under age 60, PP is not useful as a CVD risk predictor. (…) Thus, on balance, SBP is superior to PP and DBP as a way to stratify patients and as a target for treatment in older persons." [5]*
Risk assessment complexity – additional tests	*"Availability and cost considerations do not allow a widespread use of MRI in the evaluation of elderly hypertensives, but silent brain infarcts should be sought in all hypertensives with neural disturbance and, particularly, memory loss. As cognitive disturbances in the elderly are, at least in part, hypertension related, suitable cognitive evaluation tests should be used in the clinical assessment of the elderly hypertensive" [2]*<br>*"It is recommended that the ankle brachial index (ABI) be considered as a screening test for individuals with high risk for peripheral vascular disease, namely (…) age 70 years and older" [6]*
Risk management complexity – management in general	*"Use of specific drug classes in older people is largely similar to that recommended in the general algorithm and for individual compelling indications" [5]*<br>*"In the elderly, blood pressure decrease must be gradual to ensure good tolerability and guarantee a good quality of life." [7]*

	"For individuals taking any dose of statins, it is reasonable to use caution in individuals >75 years of age, as well as in individuals that are taking concomitant medications that alter drug metabolism, taking multiple drugs, or taking drugs for conditions that require complex medication regimens (…). A review of the manufacturer's prescribing information may be useful before initiating any cholesterol-lowering drug since RCTs considered defined populations and many patients in everyday practice would not qualify for clinical trials. Thus, clinicians should also consult other sources of safety data such as pharmacists, drug information centers, and manufacturers' prescribing information on a regular basis for up-to-date guidance." [8]<br>*"Suggested activities for older adults: Walking, mall walking, gardening, golfing, water aerobics, bowling, tai chi, light weight training, light house work" [9]*
Treatment targets	*"In some patients (e.g. the very elderly), recommended target levels may not be tolerable of achievable. In this case, comorbidities and individual cardiovasulcar risks should be considered when planning management." [10]*<br>*"BP goal is the same as in younger patients, i.e. 140/90 mmHg or below, if tolerated. Many elderly patients need two or more drugs to control blood pressure and reductions to 140 mmHg systolic may be particularly difficult to obtain." [2]*
Cognition and treatment adherence	*"While age has not been definitively associated with adherence, the prevalence of cognitive and functional impairments in elderly clients is known to increase their risk of poor adherence. Multiple co-morbidities and complex medical regimens further compromise adherence." [9]*<br>*"Long-acting drugs are preferred due to better patient compliance (an aspect particularly valuable in the elderly in whom a simplified drug delivery is recommended)." [7]*
**Tailoring treatment to older people context and preferences**	
Patient context and preferences	*"For patients older than 75 years of age, the Framingham model is not well validated. Though clinical studies are currently under way to address this group, at this point clinical judgement is required in consultation with the patient to determine the value of pharmacotherapy. (…)" [11]*<br>*"In adults aged over 74 years, the decision to initiate therapy should be based on clinical judgement which takes into account: likely benefits and risks of treatment, life expectancy, co-morbidities and quality of life, personal values." [3]*
Weighing benefits/harms and therapy prioritisation	*"Few data were available to indicate an ASCVD event reduction benefit in primary prevention among individuals >75 years of age who do not have clinical ASCVD. Therefore, initiation of statins for primary prevention of ASCVD in individuals >75 years of age requires consideration of additional factors, including increasing comorbidities, safety considerations, and priorities of care" [8]*
Deprescribing	*"(…) there is no reason for interrupting a successful and well tolerated therapy when a patient reaches 80 years of age" [2]*

REFERENCES

1. National Institute for Health and Clinical Excellence. NICE Clinical Guideline 67: Lipid modification - Cardiovascular risk assessment and the modification of blood lipids for the primary and secondary prevention of cardiovascular disease. National Institute for Health and Clinical Excellence , London. 2010.
2. The Task Force for the Management of Arterial Hypertension of the European Society for Hypertension (ESH) and of the European Society of Cardiology (ESC). 2007 Guidelines for the management of arterial hypertension. *Eur Heart J*. 2007;28:1462-536.
3. National Vascular Disease Prevention Alliance. Guidelines for the management of absolute cardiovascular disease risk. NVDPA, Australia. 2012.
4. The Task Force for the management of dyslipidaemias of the European Society of Cardiology (ESC) and the European Atherosclerosis Society (EAS). ESC/EAS Guidelines for the management of dyslipidaemias. *Eur Heart J*. 2011;32:1769-818.
5. U.S. Department of Health and Human Services, National Institutes of Health, National Heart, Lung, and Blood Institute, National High Blood Pressure Education Program. The Seventh Report of the Joint National Committee on Prevention, Detection, Evaluation, and Treatment of High Blood Pressure. U.S. Department of Health and Human Services. 2004.
6. Goh LG, Chua T, Kang V, Kwong KH, Lim WY, Low LP, Pereira J, Venketasubramanian N, Sethi SK, Sum CF, Tan CL, Tan HK, Tan SM, Wong TK. Ministry of Health Clinical Practice Guidelines: screening of cardiovascular disease and risk factors. *Singapore Med J*. 2011;52:220-5.
7. Sanchez RA, Ayala M, Baglivo H, Velazquez C, Burlando G, Kohlmann O, Jimenez J, Jaramillo PL, Brandao A, Valdes G, Alcocer L, Bendersky M, Ramirez AJ, Zanchetti A, Latin American Expert Group. Latin American guidelines on hypertension. *Journal of Hypertension*. 2009;27:905-922.
8. Stone NJ, Robinson J, Lichtenstein AH, Barey Mertz CN, Blum CB, Eckel RH, Goldberg AC, Gordon D, Levy D, Lloyd-Jones DM, McBride P, Schwartz JS, Shero ST, Smith SC, Watson K, Wilson PW. ACC/AHA Guideline on the Treatment of Blood Cholesterol to Reduce Atherosclerotic Cardiovascular Risk in Adults: A Report of the American College of Cardiology/American Heart Association Task Force on Practice Guidelines. *Circulation*. 2013.
9. Registered Nurses Association of Ontario. Nursing Management of Hypertension. 2005. Supplement: 2009. Heart and Stroke Foundation of Ontario, Ontario. 2005.
10. National Heart Foundation of Australia. Guide to management of hypertension 2008. Updated December 2010. National Heart Foundation of Australia. 2010.
11. Anderson TJ, Gregoire J, Hegele RA, Couture P, Mancini GB, McPherson R, Francis GA, Poirier P, Lau DC, Grover S, Genest J, Jr., Carpentier AC, Dufour R, Gupta M, Ward R, Leiter LA, Lonn E, Ng DS, Pearson GJ, Yates GM, Stone JA, Ur E. 2012 update of the Canadian Cardiovascular Society guidelines for the diagnosis and treatment of dyslipidemia for the prevention of cardiovascular disease in the adult. *The Canadian Journal of Cardiology*. 2013;29:151-67.
